# Hepatitis B Virus X Protein Stimulates Proliferation, Wound Closure and Inhibits Apoptosis of HuH-7 Cells via CDC42

**DOI:** 10.3390/ijms18030586

**Published:** 2017-03-08

**Authors:** Yongru Xu, Yingzi Qi, Jing Luo, Jing Yang, Qi Xie, Chen Deng, Na Su, Wei Wei, Deshun Shi, Feng Xu, Xiangping Li, Ping Xu

**Affiliations:** 1 State Key Laboratory of Proteomics, Beijing Proteome Research Centre, National Engineering Research Centre for Protein Drugs, National Centre for Protein Sciences Beijing, Institute of Radiation Medicine, Beijing 102206, China; kukuxong.2008@163.com (Y.X.); qi840310@foxmail.com (Y.Q.); nmluojj@126.com (J.L.); 2012283060078@whu.edu.cn (J.Y.); xieqi.tmmu@gmail.com (Q.X.); 18943956196@163.com (C.D.); sunaisthefirst@163.com (N.S.); powerweiwei@163.com (W.W.); 2 State Key Laboratory for Conservation and Utilization of Subtropical Agro-Bioresources, Guangxi University, Nanning 530005, China; ardsshi@gxu.edu.cn; 3 Key Laboratory of Combinatorial Biosynthesis and Drug Discoveryof Ministry of Education, School of Pharmaceutical Sciences, School of Basic Medical Science, Wuhan University, Wuhan 430071, China; 4 Graduate School, Anhui Medical University, Hefei 230032, China

**Keywords:** HBx, CDC42, HuH-7 cell, quantitative proteomics, hepatocellular carcinoma

## Abstract

Chronic hepatitis B virus (HBV) infection has been considered as the major cause of hepatocellular carcinoma (HCC). Hepatitis B virus X protein (HBx) has been reported to be oncogenic. The underlying mechanisms of HBV-related HCC are not fully understood, and the role played by the HBx protein in HBV induced carcinogenesis remains controversial. CDC42, a member of the Rho GTPase family, has been reported to be overexpressed in several different cancers, including HBV-related HCC. However, the specific role of CDC42 in HCC development remains unclear. Here, we investigated the cellular mechanisms by which CDC42 was responsible for the higher proliferation of HuH-7 cells mediated by HBx. We found that the expression level of CDC42 and its activity were significantly increased in HuH-7-HBx cells. The deficiency of CDC42 using the CRISPR/Cas9 system and inhibition by specific inhibitor CASIN led to the reduction of HBx-mediated proliferation. Furthermore, we observed that IQ Motif Containing GTPase Activating Protein 1 (IQGAP1), the downstream mediator of the CDC42 pathway, might be involved in the carcinogenesis induced by HBx. Therefore, the HBx/CDC42/IQGAP1 signaling pathway may potentially play an important role in HBx-mediated carcinogenesis.

## 1. Introduction

Hepatocellular carcinoma (HCC) is the fifth common cause of cancer related death worldwide [1]. Chronic hepatitis B virus (HBV) infection contributes to the great majority of HCC. Compared with the general population, chronic HBV carriers have a 5- to 15-fold increased risk of HCC [2]. Hepatitis B virus X protein (HBx) has been implicated in HBV-related hepatocarcinogenesis and is considered to be oncogenic [3,4]. HBx transgenic mice can develop HCC simultaneously, further demonstrating that HBx may have independent carcinogenic effects [5]. Therefore, the specific role of HBx in the development of HCC has attracted widespread interest.

Cell division cycle 42 (CDC42), a member of the Rho family, flips back and forth between an inactive GDP-bound form and active GTP-bound state, acting as molecular switches. CDC42 is known to contribute to tumorigenesis and cancer progression. Although there are no known activating mutations of CDC42, which result in its proto-oncogenic behavior, it is reported to be overexpressed in several different cancers [6]. Specifically, CDC42 has been shown to induce cellular transformation, invasion, and metastasis in several tissue types including melanoma, breast cancer, and colorectal cancer [7,8,9]. Furthermore, the genetic knockout of CDC42 results in cell cycle arrest and apoptosis in colorectal cancer [6]. Indeed, the analysis of CDC42 expression in 20 human liver samples revealed that compared with peri-cancerous tissues, HBV-related HCC tissues have a higher CDC42 expression [10]. CDC42 is also over-expressed at the mRNA level in HCV linked HCC than that in normal liver [11]. In our previous study, we found that the HBx-transgenic mice have different protein expression profiles compared with the wild-type mice. In addition, the lipid metabolism and CDC42-induced cytoskeleton remodeling pathways were strongly activated by the HBx transgene. However, the specific function of CDC42 in hepatocyte tumorigenicity has not been fully elucidated [12].

In this study, we found that HBx could increase the proliferative potential and cell mobility of HuH-7 cells, while decreasing the apoptotic cells. Furthermore, we detected that both the expression level and activity of CDC42 were up-regulated in HBx-expressing HuH-7 cells. Deficiency of CDC42 by the CRISPR/Cas9 (Clustered regularly interspaced short palindromic repeats) system and inhibition by the specific inhibitor CASIN significantly reduced the proliferation of HuH-7 cells promoted by HBx. Our results also implied that IQ Motif Containing GTPase Activating Protein 1 (IQGAP1), a downstream effector of CDC42, was involved in the HBx-mediated cellular proliferation. Taken together, our findings revealed that the HBx/CDC42/IQGAP1 signaling pathway might be involved in HBx-mediated higher cellular proliferation of HuH-7 cells.

## 2. Results

### 2.1. Hepatitis B Virus X Protein Promoted Proliferation and Inhibited Apoptosis of HuH-7 Cells with Up-Regulated Expression of CDC42

**Figure 1:**
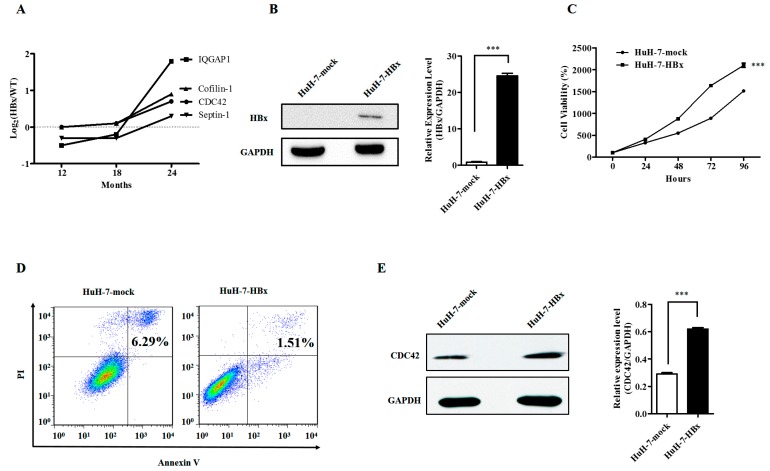
Hepatitis B virus X (HBx) protein promoted proliferation and inhibited apoptosis of HuH-7 cells with up-regulated expression of CDC42. (**A**) Up-regulated proteins associated with CDC42 as well as CDC42 in the 24-month-old HBx-transgenetic mice; (**B**) Western blot and real-time RT-PCR assays showed increased expression of HBx in HuH-7-HBx cells. *** *p* < 0.001. Data represent the mean ± SEM of three different experiments; (**C**,**D**) cell viability assay (**C**) and cell apoptosis assay (**D**) was performed in HuH-7-mock cells and HuH-7-HBx cells. *** *p* < 0.001. Data represent the mean ± SEM of three different experiments; (**E**) Western blot and real-time RT-PCR assays showed increased expression of CDC42 in HuH-7-HBx cells. *** *p* < 0.001. Data represent the mean ± SEM of three different experiments.

Our previous quantitative proteomic studies of HBx transgenic mice suggested that the CDC42 together with its downstream signaling mediators, such as IQ Motif Containing GTPase Activating Protein 1 (IQGAP1), Septin-1, and Cofilin-1 (Figure 1A), play a vital role in HBx induced carcinogenesis of mouse hepatocytes [12]. Therefore, we firstly ascertained whether HBx could promote the proliferation of HuH-7 cells. The HuH-7 cells were stably transfected with plasmid containing HBx gene, and ectopic HBx expression was confirmed by real time RT-PCR and Western blot in HuH-7-HBx cells (Figure 1B). We additionally tested whether ectopic expression of the HBx gene could enhance cellular growth. A cell viability assay was performed in HuH-7-mock and HuH-7-HBx cells to assess cell proliferation over a time course. We found that HuH-7-HBx cells had a significant increase in growth rate over HuH-7-mock cells (Figure 1C). The anti-apoptotic effect is especially critical for cancer cells, since many of them are poised on the brink of activating the cell suicide program. Therefore, a cell apoptosis analysis was performed to assess whether HBx could contribute to the anti-apoptotic capacity of HuH-7 cells. The flow cytometry results showed that HuH-7-HBx cells had a decrease in late cell apoptosis over HuH-7-mock cells (Figure 1D). Meanwhile, we found that accompanied with the expression of HBx in HuH-7 cells, the expression of CDC42 was up-regulated at both the mRNA and protein level (Figure 1E). This was consistent with the results of our quantitative proteomic studies. We therefore focused our studies on the characterization of CDC42’s role in HBx-mediated cellular proliferation and apoptosis.

### 2.2. CDC42 Was Required by HBx to Stimulate Proliferation, Wound Closure, and Inhibit Apoptosis of HuH-7 Cells

**Figure 2:**
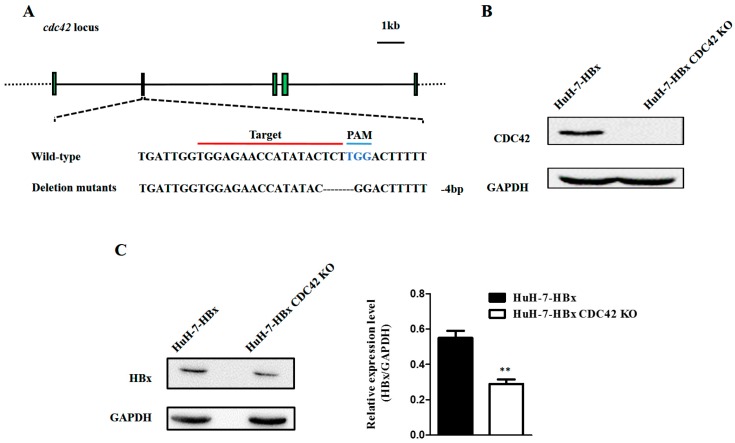
Knockout of CDC42 protein by the CRIPSR/Cas9 system. (**A**) Edited *cdc42* gene in HuH-7-HBx genome through the CRISPR/Cas9 system. In HuH-7-HBx cells, there was a mutation of 4 base deletion near the PAM (Protospacer adjacent motif) in exon2 of *cdc42* gene, which resulted in a frameshift mutation; (**B**,**C**) Western blot analysis for the expression of CDC42 (**B**) and HBx (**C**) in the HuH-7-HBx and HuH-7-HBx CDC42 KO cells. ** *p* < 0.01. Data represent the mean ± SEM of two independent experiments.

**Figure 3:**
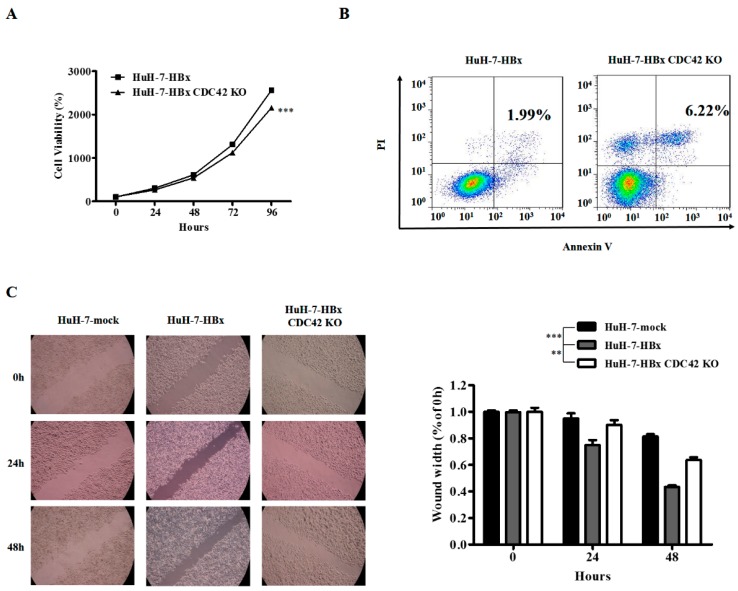
CDC42 was required by HBx to stimulate proliferation, wound closure, and inhibit apoptosis of HuH-7 cells. (**A**) The proliferation rate of HuH-7-HBx and HuH-7-HBx CDC42 KO cells was measured over 4 days. *** *p* < 0.001. Data represent the mean ± SEM of three different experiments; (**B**) cell apoptosis of HuH-7-HBx and HuH-7-HBx CDC42 KO cells was measured by flow cytometry; (**C**) analysis of cell migration in HuH7-mock, HuH-7-HBx, and HuH-7-HBx CDC42 KO cells, as measured by a wound-healing assay. Left: Representative photographs taken at 0, 24, and 48 h post-wound (×20). Right: The wound closure was quantified at 0, 24, and 48 h post-wound by measuring the remaining unmigrated area using ImageJ. ** *p* < 0.01. *** *p* < 0.001. Data represent the mean ± SEM of two different experiments.

To determine the role of CDC42 in HBx-mediated cellular proliferation and apoptosis, the HuH-7-HBx cells was transfected with gRNA1-CRISPR/Cas9 plasmid (Figures S1–S3), and stable sub-clonal populations were selected and cultured. The sequencing results showed that HuH-7-HBx CDC42 KO (knock out) cells had a mutation of 4 base deletion at the *cdc42* locus (Figure 2A and Figure S4). Knockout of CDC42 was confirmed through Western blot (Figure 2B), and the expression of HBx was decreased in HuH-7-HBx-CDC42 KO cells (Figure 2C). We then examined the cellular phenotypic effects after knockout of CDC42. A cell viability assay was also performed in HuH-7-HBx and HuH-7-HBx CDC42 KO cells to estimate cell proliferation over a time course. These results showed that HuH-7-HBx CDC42 KO cells had a significant decrease in cell proliferation over wild type HuH-7-HBx cells (Figure 3A). Expectedly, the anti-apoptotic effect mediated by HBx had been repressed when CDC42 was absent (Figure 3B). CDC42, as a member of the Rho-GTPase family, plays a vital role in the processes of cellular movement and migration through its regulation of cytoskeletal changes. A wound healing assay revealed that HuH-7 cells’ migration capacity was notably enhanced by HBx. When CDC42 was absent, the enhanced migration capacity of HuH-7-HBx cells was attenuated partially (Figure 3C). Taken together, these data indicated that CDC42 was required by HBx to stimulate proliferation, wound closure, and inhibit apoptosis of HuH-7 cells.

### 2.3. CDC42 Inhibitor Reduced Proliferation and Promoted Apoptosis Only in HBx-Expressing HuH-7 Cells

**Figure 4:**
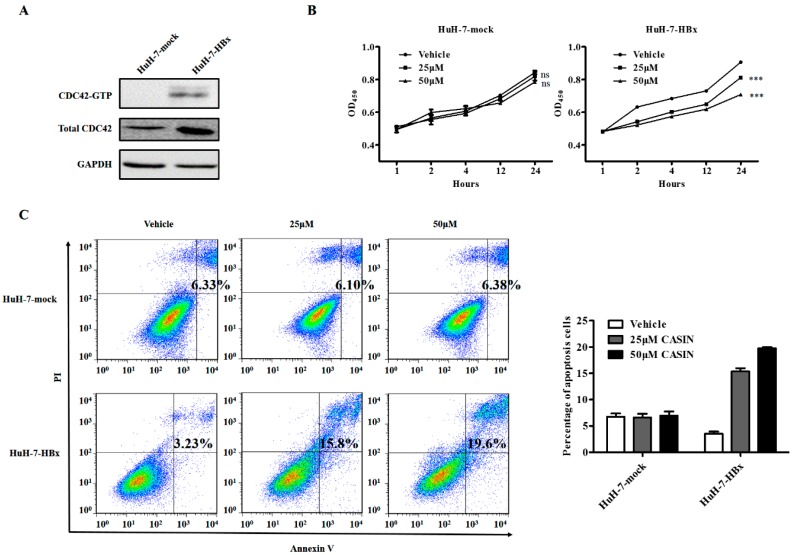
CDC42 inhibitor reduced proliferation and promoted apoptosis only in HBx-expressing HuH-7 cells. (**A**) Active CDC42 was detected by the pull-down assay using GST-PAK1-PBD beads in HuH-7-mock and HuH-7-HBx cells; (**B**,**C**) cell viability assay (**B**) and Cell apoptosis analysis (**C**) was conducted in HuH-7-mock and HuH-7-HBx cells cultured without or with treatment of different doses of CASIN, implying that HuH-7-HBx cells was more sensitive to CDC42 inhibition. *** *p* < 0.001. Data represent the mean ± SEM of two different experiments. ns, not significant.

The wound healing assay suggested that HBx may target the CDC42 signaling pathway in promoting migration of HuH-7 cells. To examine whether the effect of HBx on HuH-7 cell migration was mediated through CDC42 at the biochemical level, we analyzed CDC42 activation by using a GST pull-down assay. In this assay, GTP-bound active CDC42 (CDC42-GTP) was selectively retained by binding to the Pak1-binding domain (PBD) of its substrate Pak1 in the GST-Pak1 PBD matrix. The results showed that HuH-7-HBx cells had a higher level of CDC42-GTP (Figure 4A) indicating that the expression of HBx stimulated formation of CDC42-GTP complexes (active). CASIN, a novel CDC42 Activity-Specific Inhibitor [13,14], that is effective in suppressing CDC42 activity, was employed to treat HuH-7-mock and HuH-7-HBx cells. After treatment of CASIN, a cell viability assay revealed that CASIN significantly inhibited the growth of HuH-7-HBx cells in which the expression of CDC42 was up-regulated, but had little effect on the proliferation of HuH-7-mock cells (Figure 4B). As for the anti-apoptotic effect, similarly, when CDC42 activity was suppressed in HuH-7-HBx cells, the anti-apoptotic effect mediated by HBx was also suppressed in a dose-dependent manner, while there was nearly no change in HuH-7-mock cells (Figure 4C). The above results indicated that HuH-7-HBx cells retained a higher level of activated CDC42, resulting in HuH-7-HBx cells being more sensitive to CASIN. The specific inhibition of CDC42 partially attenuated the phenotype of HuH-7 cells caused by HBx, implying that CDC42 activity would contribute to the HBx-mediated proliferation and apoptosis.

### 2.4. Quantitative Proteomics Implied That IQGAP1 Participated in the HBx-Mediated Proliferation of HuH-7 Cells

**Figure 5:**
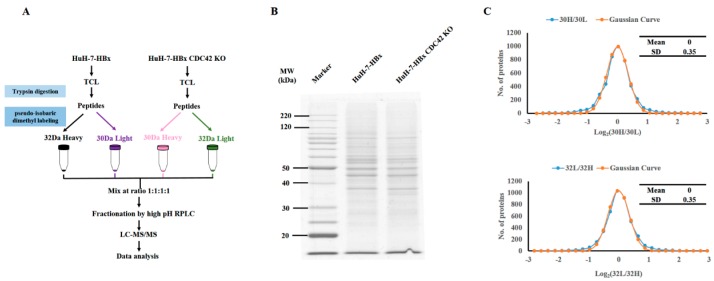
Quantitative proteomics strategies and acquisition of high quality proteomic data. (**A**) Experimental procedures of the quantitative proteomic analysis in this study. TCL, total cell lysate; (**B**) Samples were resolved by SDS-PAGE and stained with Coomassie blue, indicating that a high quality and an equal starting amount of protein was prepared; (**C**) Distributions of Log_2_ (30H/30L) were symmetrical and could be fit to a Gaussian curve, and the value of Standard Deviation (SD) was 0.35, the same as that of Log_2_ (32L/32H).

To detect the downstream signal mediators regulated by CDC42, we then performed quantitative proteomics using pseudo-isobaric dimethyl labeling (pIDL) strategies to identify the molecular change when CDC42 was absent; the experimental procedure is depicted in Figure 5A. To differentiate the technical variations and determine the true biological relevance, we established technical duplicates of both HuH-7-HBx and HuH-7-HBx CDC42 KO cells. Each individual proteome sample was split into two identical aliquots to determine the variation during pseudo-isobaric labeling processes. A high quality of the protein sample and the same starting material were required to perform a reliable quantitative comparison. We resolved these samples by SDS-PAGE and stained with Coomassie blue, and the results indicated that a high quality of the proteomic samples was prepared (Figure 5B).

The distributions of the ratios of Log_2_ (30H/30L) were symmetrical and could be fit to a Gaussian curve. The value of Standard Deviation (SD) of the curve was 0.35, which was the same as that of the ratios of Log_2_ (32L/32H), indicating a high reproducibility and a well-controlled experimental procedure during our proteomics studies (Figure 5C).

**Figure 6:**
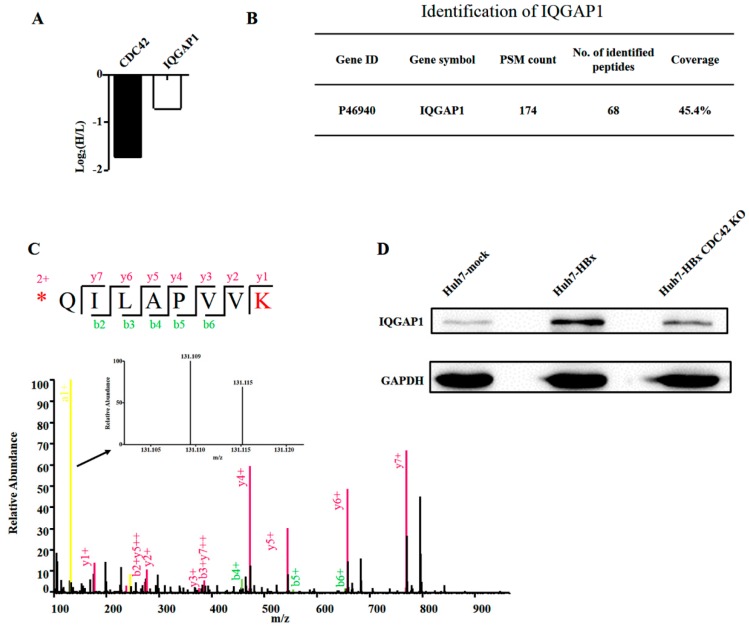
The expression of IQGAP1 was down-regulated when CDC42 was absent. (**A**) Quantitative proteomics data suggested that CDC42 and IQGAP1 were significantly down-regulated in HuH-7-HBx CDC42 KO cells; (**B**) Identification details of IQGAP1; (**C**) Verification of the quantified IQGAP1 by randomly choosing an MS_2_ spectrum and comparing the intensity of the a1 ion; (**D**) Western blot assay was performed to detect the expression of IQGAP1 in HuH-7-mock, HuH-7-HBx, and HuH-7-HBx CDC42 KO cells.

In the HuH-7-HBx (30L) and HuH-7-HBx CDC42 KO (30H) samples, in total 5662 proteins were identified. Among them, 4436 proteins were quantified. To identify significantly changed proteins, the threshold for significantly up- and down-regulated proteins in this study was set at fold change >1.5 or fold change <0.67 together with a *p*-value < 0.05. Compared with HuH-7-HBx cells, we found 523 differentially expressed proteins (Table S4) in HuH-7-HBx CDC42 KO cells, including 239 up-regulated proteins and 284 down-regulated proteins (see Supplemental Materials) after stringent filtering. Among the down-regulated proteins, we noted that accompanied with the knockout of CDC42, there appeared to be a sharp decrease of IQGAP1 (Figure 6A). We further checked the sequence coverage of IQGAP1, as illustrated in Figure 6B, and we identified 174 spectra for 68 peptides from tryptic IQGAP1. The sequencing coverage reached 45.4%, convincing the identification of IQGAP1 in our study. We also validated the quantification of IQGAP1 by checking the spectrum randomly. As illustrated in Figure 6C, the spectrum of peptide sequence QILAPVVK had a higher base peak intensity and continuous *b*/*y* ion matches. Furthermore, the a1 ion intensity of 30H samples was significantly lower than that of 30L samples, confirming that IQGAP1 was truly down-regulated in HuH-7-HBx CDC42 KO cells. Furthermore, we found that the expression of IQGAP1 was up-regulated when HBx was ectopically expressed in HuH-7 cells and down-regulated when CDC42 was absent in HuH-7-HBx cells (Figure 6D).

**Figure 7:**
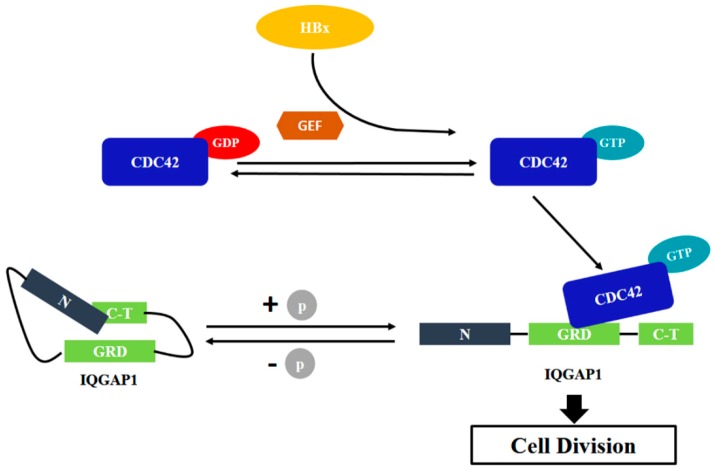
Potential schematic of the HBx-mediated effect of HuH-7 cells. In HBx-expressing HuH-7 cells, both the expression and activity of CDC42 were up-regulated. The accumulated active CDC42 interacted with IQGAP1, resulting in the dysregulation of cell proliferation, which finally caused higher proliferation of HuH-7 cells. GEF, guaninenucleotide exchanging factors; GRD, GTPase-activating protein (GAP)-related domain.

The C-terminal of IQGAP1 had been reported to interact with CDC42 to play a key role in cell division. In the present study, our proteomic data implied that IQGAP1 might interact with CDC42 to contribute to HBx-meditated proliferation of HuH-7 cells. Based on these preliminary data, we hypothesized that HBx enhanced the activity of CDC42 by interacting with the up-regulators of CDC42, such as GEFs (guaninenucleotide exchanging factors) and Rho GDIs (GDP dissociation inhibitors). The accumulated level of active CDC42 could interact with IQGAP1 to stimulate the cell division, which successively promoted the proliferation of HuH-7 cells (Figure 7).

## 3. Discussion

Chronic hepatitis B virus (HBV) infection has been considered as the major risk cause of HCC. The X protein (HBx) encoded by HBV is believed to be the major player in the process of HBV-induced oncogenesis [3,4]. It has been well documented that HBx induces tumors in transgenic animals [5,15]. However, the precise mechanisms underlying HBx-mediated hepatocarcinogenesis remain an unsolved mystery. Herein, we found that the HBx-expressing HuH-7 cells had higher proliferative, mobilized, and anti-apoptotic capacity than wild type HuH-7 cells. Consistent with previous reports that HBx has independent oncogenic effects, the deficiency of CDC42 using the CRISPR/Cas9 system reduced the proliferation mediated by HBx and the inhibition of CDC42 activity using the specific inhibitor CASIN suppressed the proliferative and anti-apoptotic potential of HuH-7-HBx cells. Furthermore, we detected that IQGAP1 might be involved in HBx-mediated proliferation by quantitative proteomic studies. In summary, we hypothesized that HBx promoted the proliferation of HuH-7 cells via the HBx/CDC42/IQGAP1 axis.

HBx is reported as a multifunctional viral protein that regulates cell proliferation, differentiation, cell cycle regulation, autophagy, and apoptosis [16,17]. Many studies have tried to explore the role of HBx in cell apoptosis and its contribution to HBV-associated HCC. The results are controversial and need to be interpreted carefully; HBx has been shown to induce [18], inhibit [19], or have no effect on apoptosis [20]. The discrepancy of the role of HBx on apoptosis may be due to the different cell types, culture conditions, or experimental designs. In the present study, we found that HBx played an anti-apoptosis role in HuH-7 cells.

Small GTPases, acting as molecular switches, can regulate the activation of multiple downstream effectors [21,22,23,24]. Among their pleiotropic actions, Rho-dependent signaling cascades modulate cellular morphology and actin polymerization, adhesion, cell migration, proliferation, and apoptosis [25,26]. As a member of the Rho-GTPases family, CDC42 has been well studied as a main regulator of cytoskeletal architecture [26,27]. It participates in multiple signaling pathways, including tyrosine kinase receptors, heterotrimeric G-protein coupled receptors, cytokine receptors, integrin, as well as physical and chemical stress [28]. CDC42 is highly expressed in various types of human cancers, such as melanoma, breast, colon cancer, as well as hepatocellular carcinoma. It has been demonstrated that CDC42 plays an important role in promoting hepatocyte proliferation and liver regeneration [29]. Van Hengel et al. showed that the hepatic deletion of CDC42 induced chronic liver damage, jaundice, and fibrosis [30], which suggested that chronic liver damage and toxic effects of bile acids induced cancer formation in CDC42-deficient mice. Thus, the function of CDC42 in HCC still needs to be explored. Expression profiling reveals dysregulation of cellular cytoskeletal genes in HBx-induced hepatocarcinogenesis [31]. The cellular cytoskeleton is mainly regulated by the Rho family of small GTP-binding proteins, CDC42, Rac1, and RhoA. CDC42 and Rac1 activation have been linked to increased HIV-1 replication [32]. In our study, we examined the expression of CDC42 in the context of ectopic expression of HBx in HuH-7 cells. Interestingly, we provided evidence that both the expression level and activity of CDC42 were significantly increased in HuH-7-HBx cells, indicating that CDC42 was implicated in the higher proliferation mediated by HBx.

To further understand the underlying mechanisms of HBx-mediated higher proliferation in HuH-7 cells, we tried to explore the downstream mediators of CDC42 employing quantitative proteomics method. Luckily, we found that accompanied with knockout of CDC42, there was a significant decrease in the expression of IQGAP1. Thus, we speculated that IQGAP1 might be involved in the HBx-mediated proliferation. IQGAPs are an evolutionary conserved family containing multi-domain proteins, which play vital roles in regulating distinct cellular processes including cell adhesion, cell migration, extracellular signaling, and cytokinesis. IQGAP1 was the first of three human IQGAP (IQGAP1/2/3) homologues discovered [33] and ubiquitously expressed. IQGAP1 could impair the intrinsic GTPase activity of CDC42, thus stabilizing CDC42 in its activated form [34]. IQGAP1 was detected to be highly expressed in several cancer types, and resulted in poor prognosis and survival of patients [35,36,37]. As an effector of CDC42, IQGAP1 can bind directly to CDC42 and has been implicated in the modulation of cell architecture and regulation of exocytosis in human cancers. It has been reported that IQGAP1 has a crucial role in transducing CDC42 signaling to the cytoskeleton [38]. IQGAP1 could serve as a phosphorylation-sensitive conformation switch to regulate the coupling of cell growth and division through a CDC42-mTOR pathway. Dysregulation of this conformation switch generated cellular transformation [39]. We previously found that the expression of IQGAP1 was up-regulated in HBx transgenetic mice compared with wild type mice. In the present study, the expression of IQGAP1 was also up-regulated in HuH-7-HBx cells compared with HuH-7-mock cells. Therefore, it is plausible to assume that IQGAP1 contributes to higher proliferation mediated by HBx in HuH-7 cells, in spite of the fact that the precise molecular mechanisms need to be further determined. This study firstly indicated that the HBx protein promoted proliferation of HuH-7 cells via CDC42.

## 4. Materials and Methods

### 4.1. Cell Culture, Cell Lines, and Reagents

HuH-7 cell, obtained from the Cell Bank of the Chinese Academy of Sciences (Shanghai, China), were cultured in RPMI 1640 medium or in DMEM supplemented with 10% fetal bovine serum, 1% l-glutamine, 100 units/mL penicillin, and 100 µg/mL streptomycin at 37 °C in 5% CO_2_. To guarantee cell line authenticity, the cell line was aliquoted and banked, and cultures were grown and used for a limited number of passages before starting a new culture from stock. Cell lines were routinely tested for mycoplasma contamination. When the cell fusion rate reached 80%, in the presence of the liposome Lipofectamine 2000 according to the manufacturer’s instructions, HuH-7 cells were transfected with plasmid pcDNA3.1-HBx, in which the full length HBx sequence was constructed in the mammalian expression vector pcDNA3.1 (Invitrogen, Carlsbad, CA, USA) as described previously [32]. Forty-eight hours post-transfection, the transfected cells were incubated in a selection medium containing 800 mg/mL G418. Stable cell lines, HuH-7-HBx cells were selected after the formation of resistant clones. The HuH-7-HBx CDC42 KO cells were maintained in the culture media supplied with 1 μg/L puromycin. CASIN (C_20_H_22_N_2_O) (Adooq, Nanjing, China) is a specific CDC42 inhibitor. 2 mg CASIN was dissolved in 1 mL Dimethyl Sulphoxide (DMSO), then diluted by culture medium to 25 and 50 μM, respectively. The control groups were treated with the same volume of DMSO, while the experimental groups were treated with 25 and 50 μM CASIN, respectively.

### 4.2. Cell Viability Assay

To determine the growth rate of different cells, cells (2 × 10^3^ cells/well) were seeded into a 96-well plate in triplicate. The cell growth rate was measured every 24 h by using the Cell Counting Kit-8 (CCK-8, Dojindo Laboratories, Kumamoto, Japan). Briefly, CCK-8 reagent (10 μL) was added into each well, incubated for 30 min at 37 °C, and subjected to absorbance measurement at 450 nm. For CDC42 specific inhibitor (CASIN) treatment experiments, cells (2 × 10^3^ cells/well) were cultured in culture media in the absence or presence of 25 or 50 μM CASIN, respectively. The cell growth rate was detected using CCK-8 at different time points (1, 2, 4, 12, and 24 h) as indicated.

### 4.3. Cell Apoptosis Assay

For cell apoptosis analysis by flow cytometry, cells were cultured until they reached a confluence of 70%–80%, trypsinized, and harvested by centrifuge. After washing with PBS two times, 1 × 10^6^ cells were suspended in 1× AnnexinV Binding Buffer, dispersed into single cells by pipetting. Taking out 100 μL of the cell suspension, 5 μL Annexin V FITC and 5 μL PI (Propidium Iodide) solution were added, and the solution was incubated at room temperature away from light for 15 min. Cells were suspended in 400 μL 1× Annexin V Binding Buffer, and analyzed by flow cytometry. To analyze the apoptosis level of cells treated with CASIN, cells (2 × 10^5^ cells/well) were seeded into 24-well plate in triplicate incubated with the culture media at 37 °C in a humidified atmosphere of 5% CO_2_ overnight. Replenished culture media was used with or without CASIN. After 12 h, the cells were harvested and treated with reagents supplied by the Annexin V, FITC Apoptosis Detection Kit (Dojindo Laboratories, Kumamoto, Japan) according to the protocol as described previously.

### 4.4. Wound Healing Assay

For the wound healing assay, cells (10^6^ cells/well) were seeded into a 6-well plate in triplicate. Cells were cultured until the confluence of 70%–80%. Cell monolayers were wounded by plastic tips. Wounded monolayers were then washed two times by PBS in order to remove cell debris. The cells were further incubated in DMEM. Cell migration and the average distance of migrating cells were observed at various time points.

### 4.5. Quantitative Real-Time RT-PCR

The total RNA of HuH-7-mock and HuH-7-HBx cells was extracted using Trizol reagent (Invitrogen, Carlsbad, CA, USA) respectively. The total RNA was then digested using RQ1 RNase-free DNase (Promega, Wisconsin, WI, USA), and reverse transcripted using the TransScript First-Strand cDNA Synthesis Super Mix (TransGen Biotech, Beijing, China), according to the protocols provided by the manufacturer. RT-PCR was performed using the Step One Plus machine (Life Technology, Carlsbad, CA, USA) with SYBR^®^ Green Real time PCR Master Mix (TOYOBO, Shanghai, China), according to the protocol provided by the manufacturer. The primers used to assess the expression of HBx and GAPDH, respectively, are shown in Table S1.

### 4.6. CRISPR/Cas9 System

The gRNAs (Tables S2 and S3) were cloned to the p-CAG-T7 vector (Vsolid, Shenzhen, China) according to the manufacturer’s guideline. Cells in 6-wells plates were maintained in 1 mL culture media and transfected with 1.5 μg of plasmid containing gRNA1 together with 1.5 μL of transfection reagent. Cells were incubated at 37 °C for 24 h, before adding culture media containing 1 μg/mL puromycin. The cell culture media was gently replenished every 24 h from then on. One week later, the cells were trypsinized and harvested for limiting dilution assay, as previously described [40].

### 4.7. DNA Extraction

The genomic DNA was extracted by using a general type genomic DNA extraction kit (CWBIO, Shanghai, China), according to the protocol provided by the manufacturer. Briefly, pelleted cells were resuspended in 200 μL GTL solution, 20 μL of Proteinase K and 200 μL of GL were added, and the cells were incubated at 56 °C for 10 min. Then, 500 μL of GW1 solution and 500 μL of GW2 solution was added to the reaction system. Finally, the genomic DNA was dissolved in 30 μL ddH_2_O.

### 4.8. Western Blot Analysis

Cells were harvested at the confluence of 70%–80%, pelleted cells were treated with lysis buffer, ultra-sonicated for 8 min, and centrifuged at the highest speed at 4 °C for 12 min. The CDC42 antibody (Santa Cruz, CA, USA) was used to detect the expression of CDC42. To detect active CDC42, CDC42-GTP was pulled down using GST-PAK1-PBD beads provided by the CDC42 Pull-down Activation Assay Biochem Kit (Cytoskeleton, Denver, CO, USA), according to the protocol provided by the manufacturer. GAPDH protein was detected by using the GAPDH antibody (CWBIO, Shanghai, China). Densitometric analysis was carried out using ImageJ software.

### 4.9. Quantitative Proteomics Analysis

Approximately 1 × 10^7^ cells were harvested by centrifuge and washed by 1× PBS three times. The cells were further homogenized in 350 μL lysis buffer (1% (*v*/*v*) protease inhibitor cocktail in 8 M urea), ultra-sonicated for 8 min, and ultra-centrifuged at the highest speed for 12 min at 4 °C. Taking out 100 μg protein from each sample, they were treated with 5 mM DTT for reduction and 20 mM iodoacetamide (IAA) for alkylation, then precleared by SDS-PAGE (10%, 0.5 cm). The samples were digested in gel using trypsin at a concentration of 10 ng/mL, incubated at 37 °C for 14 h, and extracted using extraction buffer (5% formic acid, 50% acetonitrile (ACN)). The HuH-7-HBx digests were labeled with ^13^CH_2_O and NaCNBH_3_ (light labeling, 30L), the HuH-7-HBx CDC42 KO digests were labeled with CH_2_O and NaCNBD_3_ (heavy labeling, 30H), HuH-7-HBx digests were labeled with CD_2_O and NaCNBH_3_ (heavy labelling, 32H) and the HuH-7-HBx CDC42 KO digests were labeled with ^13^CH_2_O and NaCNBD_3_ (light labeling, 32L), as previously described [41]. Next, the four differently labeled digests were mixed at a 1:1:1:1 ratio.

The peptide mixtures were loaded onto a Durashell C18 column (150 Å, 3 μm, 2.1 × 100 mm^2^). Briefly, the solvent gradients of buffer A (98% ddH_2_O and 2% ACN, pH 10, adjusted by ammonium hydroxide) and buffer B (2% ddH_2_O and 98% ACN, pH 10) were as follows: 2% B, 3 min; 5% B, 3 min; 22% B, 34 min; 35% B, 15 min; 85% B, 1 min; 85% B, 2 min and 3% B, 2 min. The LC flow rate was set at 0.1 mL/min and monitored at 214 nm. The column oven was set at 45 °C. In our experiments, we used a 2-min collection interval, 30 fractions were accepted, and finally according to the results of the chromatographic fractions we merged the 30 fractions into 10 tubes, then dried and suspended them with loading buffer containing 0.1% FA and 1% ACN before LC−MS/MS analysis.

The second-dimension analysis was performed on low-pH RP (Reverse Phase) chromatography separation coupled with tandem mass spectrometry (LC−MS/MS) using an Q Exactive HF mass spectrometer (Thermo Electron, San Jose, CA, USA). The instrument was interfaced to an ultra-performance liquid chromatography (UPLC) system (Ultimate 3000, Thermo Fisher, Waltham, MA, USA). The labeled peptide mixtures were loaded onto a 150 μm i.d. × 12 cm fused-silica capillary column (Beijing 200 SpectraPeaks, Beijing, China) packed with C18 resins (120 Å; 3 μm; Michrom Bioresources, Inc., Auburn, CA, USA) with a flow rate of 0.6 μL/min and monitored at 214 nm. Briefly, the solvent gradients of buffer A (ddH_2_O and 0.1% Formic acid) and buffer B (ACN and 0.1% Formic acid) were as follows: 9% B, 16 min; 12% B, 36 min; 23% B, 15 min; 31% B, 7 min; 95% B, 7 min. The MS parameters were as follows. Eluted peptides were ionized under high voltage (2.2 kV) and analyzed using an orbitrap mass spectrometer (Thermo Fisher, Waltham, MA, USA) in a survey scan (300–1400 *m*/*z*). The MS1 pre-cursors were detected in the centroid mode, and the resolution was set at 6 × 10^4^ at *m*/*z* with the accumulation of automatic gain control (AGC) target reaching up to 3 × 10^6^ under the limitation of the 80 ms maximum ion injection time (MIT). The twenty most intense ions were selected for further fragmentation in the data-dependent mode via higher energy collision induced dissociation (HCD) with 40% collision energy. The MS_2_ fractions were detected in the orbitrap using the profile mode with the lowest recorded mass fixed at 100 *m*/*z*, and at a resolution of 3 × 10^4^ at *m*/*z*. The isolation window was operated at 2.0 *m*/*z*, and AGC was set as 2 × 10^5^ accumulated in the linear ion trap. The dynamic exclusion was set as 12 s for avoiding the redundancy detections.

The acquired raw files were then submitted to the pFind 3.0 search engine against the Uniprot human reference protein database (version November 2015). The search parameters were as follows: precursor ion spectra were searched with a search tolerance of 20 ppm in the MS mode, and the MS_2_ ions with 20 ppm in the HCD mode. Heavy- and light-labeled samples were searched independently using dimethyl (+30.03801 Da) and dimethyl (+30.04385 Da) N-termini and K as variable modifications. The peptides and proteins were filtered to a FDR (False Discovery Rate) of lower than 1%. Protein quantification was based on the MS_2_ Ratio a1.

### 4.10. Statistical Analysis

Data were analyzed using GraphPad Prism software or Excel, and expressed as means ± SEM. Statistical significance for paired samples and for multiple comparisons was determined using Student’s *t* test and one-way ANOVA with Tukey’s test, respectively. Data were considered statistically significant at *p* < 0.05.

## Supplementary Materials

Supplementary materials can be found at www.mdpi.com/1422-0067/18/3/586/s1.
