# Supplementary material for: Hepatitis B Virus X Protein Stimulates Proliferation, Wound Closure and Inhibits Apoptosis of HuH-7 Cells via CDC42
Source: Int J Mol Sci. 2017 Mar 8;18(3):586. doi: 10.3390/ijms18030586 (PMC5372602; doi:10.3390/ijms18030586)
Supplement: Supplementary file 1 [file ijms-18-00586-s001.zip › ijms-177668-supple-proof reading.pdf]

Supplemental Data Legends

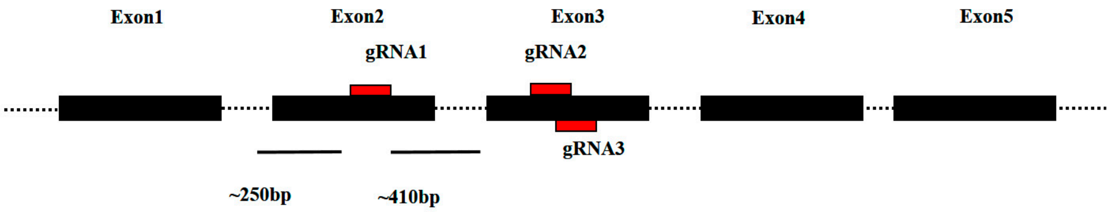

Figure S1. The target sequence of CRISPR/Cas 9 in CDC42 locus. We completely designed 3 pairs of gRNAs, one of which targeted at exon2, and the others which targeted at exon3.

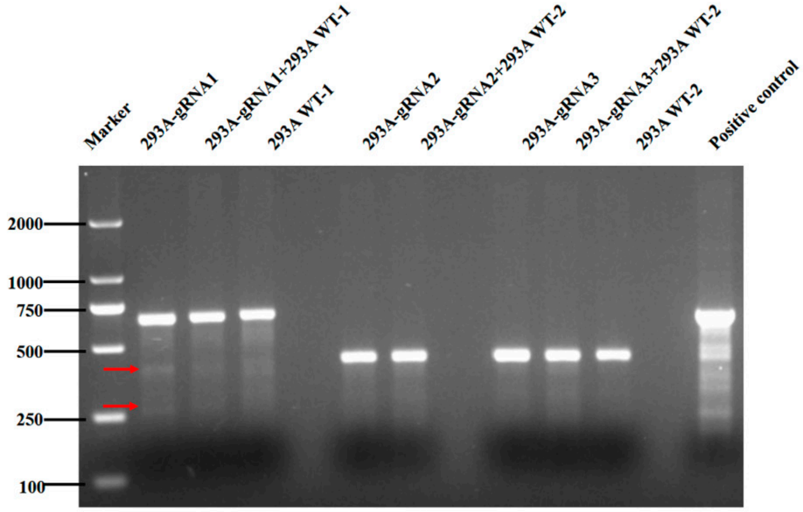

Figure S2. The T7E1 assay was used to detect the endogenous shear activity of gRNAs in 293A cells. 293A Cells were transected with gRNA1/2/3-Cas9 plasmids, respectively, and cultured for 2 days. Mutation frequencies were measured using the T7E1 assay. 500 ng PCR-amplified fragments from genomic DNA were used and generated bands of the expected sizes in targeted vs. non-targeted individuals.

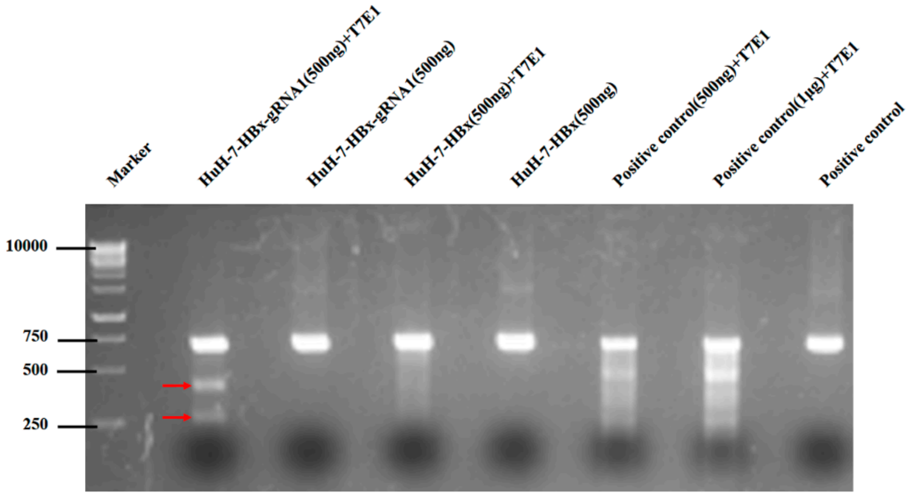

Figure S3. The T7E1 assay was used to detect the endogenous shear activity of gRNA1 in HuH-7-HBx cells. HuH-7-HBx cells were transfected with plasmids containing gRNA1-Cas9 and cultured for 2 days. Mutation frequencies were measured using the T7E1 assay.

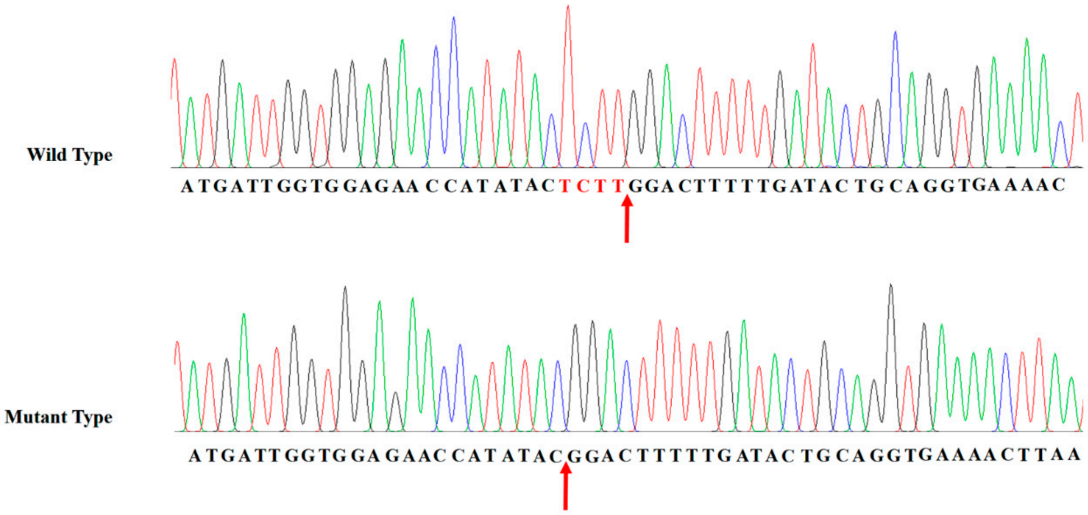

Figure S4. Chromatograms of DNA sequences of wild-type cells and edited cells obtained in this study. Chromatogram for the introduction of a 4 bp deletion within CDC42 ORF.

Table S1. The primers of HBx and GAPDH gene for RT-PCR.

Gene name	Oligonucleotide sequence
HBx	F:5′-GTCTCCTCTGACTTCAACAGCG-3′
	R:5′-ACCACCCTGTTGCTGTAGCCAA-3′
GAPDH	F:5′-CCGTCTGTGCCTTCTCATCTGC-3′
	R:5′-CAAGGTCGGTCGTTGACATTGC-3′

Table S2. Oligonucleotide sequence of CDC42 Exon2/3-gRNA.

gRNA	Oligonucleotide sequence
CDC42-Exon2-gRNA-1	F: 5′ -TGGAGAACCATATACTCT***TGG***-3′
	R: 5′ -CCAAGAGTATATGGTTCTCCA-3′
CDC42-Exon3-gRNA-1	F: 5′ -TGTTTGTGGATAACTCAG***CGG***-3′
	R: 5′ -CCGCTGAGTTATCCACAAACA-3′
CDC42-Exon3-gRNA-2	F: 5′ -TAGAAATACATCTGTTTG***TGG***-3′
	R: 5′ -CCACAAACAGATGTATTTCTA-3′

Table S3. Monoclonal gene identification primer.

Primer	Oligonucleotide sequence
CDC42-g1-F	5′ -GCAGAAATCTCAGAACACATCC-3′
CDC42-g1-R	5′ -GGGAGAGAATCAGTGGGCA-3′
CDC42-g2/3-F	5′ -CACTTTGAGGACACCAAGATTC-3′
CDC42-g2/3-R	5′ -GCAGTCTCTGGAGTGATAGGCT-3′
